# bHLH Family Transcription Factors: Molecular Switches in Plant Specialized Metabolism

**DOI:** 10.3390/cells15151400

**Published:** 2026-08-03

**Authors:** Xinpei Han, Guodong Chen, Jun Peng, Nan Cao, Fuguang Li, Sumei Wan

**Affiliations:** 1 College of Agriculture, Tarim University, Alar 843300, China; hnndhxp921230@163.com (X.H.); guodongchen@taru.edu.cn (G.C.); caonan@taru.edu.cn (N.C.); 2 National Nanfan Research Institute, Chinese Academy of Agricultural Sciences, Sanya 572024, China; jun_peng@126.com; 3 Institute of Cotton Research, Chinese Academy of Agricultural Sciences, Anyang 455000, China

**Keywords:** bHLH transcription factor, MYC2, jasmonate, specialized metabolism

## Abstract

**Highlights:**

**What are the main findings?**

- Plant bHLH factors regulate specialized metabolism through direct, cascade, or hybrid architectures centered on signal-responsive modules such as JA-JAZ-MYC.
- Cross-species comparison identifies six determinants of bHLH output: signal gating, pathway topology, promoter and partner logic, spatial competence, feedback regulation, and evidence context.

**What are the implications of the main findings?**

- Effective metabolic engineering should match the intervention to the pathway bottleneck and combine transcription-factor tuning with tissue-specific control, precursor supply, transport, and storage capacity.
- This framework supports more precise improvement of crop defense, food quality, and medicinal-metabolite production while minimizing growth penalties and toxicity.

**Abstract:**

Plant specialized metabolites connect genetic programs and environmental responses with ecologically and economically valuable natural products. Their accumulation is rarely constitutive, varying instead with tissue identity, developmental stage, stress exposure, hormone signaling, and cellular storage capacity. This review examines basic helix-loop-helix (bHLH) transcription factors as regulatory switch points in plant specialized metabolism, with emphasis on the jasmonate-JAZ-MYC module. In resting tissues, JAZ repressors constrain MYC/bHLH activity; after wounding, herbivory, pathogen challenge, or elicitation, jasmonoyl-isoleucine triggers COI1-dependent JAZ turnover, releasing MYC factors to bind E-box/G-box motifs, recruit coregulators such as MED25, and activate biosynthetic genes or downstream transcription-factor cascades. Plant lineages have repeatedly adapted this regulatory logic to control terpenoids, alkaloids, phenylpropanoids, flavonoids, glucosinolates, phytoalexins, and related metabolites. Comparative examples include Arabidopsis sesquiterpenes and glucosinolates, Taxus taxanes, Artemisia artemisinin, Catharanthus terpenoid indole alkaloids, Salvia phenolic acids and tanshinones, Ginkgo terpene trilactones, rice diterpenoid phytoalexins, and cotton gossypol. Across these systems, bHLH output depends on dimer choice, promoter grammar, chromatin accessibility, hormone crosstalk, partner transcription factors, and cell-type competence. Six shared principles emerge: signal gating, topology matched to pathway architecture, partner-dependent promoter decoding, spatial competence, feedback rheostats, and evidence-dependent transferability. We further discuss evidence standards, multi-omics-guided factor discovery, miRNA-mediated post-transcriptional control, and engineering strategies for crop defense, food quality, medicinal-metabolite production, and synthetic biology. Unlike pathway- or MYC2-centered surveys, this review organizes the literature within a direct–cascade–hybrid framework that integrates promoter grammar, spatial competence, storage anatomy, and an explicit evidence hierarchy.

## 1. Introduction

Plant specialized metabolism forms a major component of the chemical interface between plants and their environments. Although many specialized metabolites are dispensable for basal growth under protected conditions, they contribute to herbivore and pathogen resistance, ecological communication, abiotic-stress tolerance, and traits valued in agriculture, nutrition, and medicine. These compounds—including terpenoids, alkaloids, phenylpropanoids, flavonoids, glucosinolates, quinones, phytoalexins, and others—differ widely in structure and biosynthetic origin, yet share a defining feature: their accumulation is conditional. Pathway output depends not only on biosynthetic-enzyme abundance but also on developmental stage, tissue identity, hormone status, prior stress exposure, precursor supply, transport, and storage capacity. Specialized metabolism is therefore better understood as a regulated cellular program than as a static inventory of biosynthetic reactions [1,2,3,4,5,6,7,8,9,10,11,12,13,14,15,16,17].

**Figure 1:**
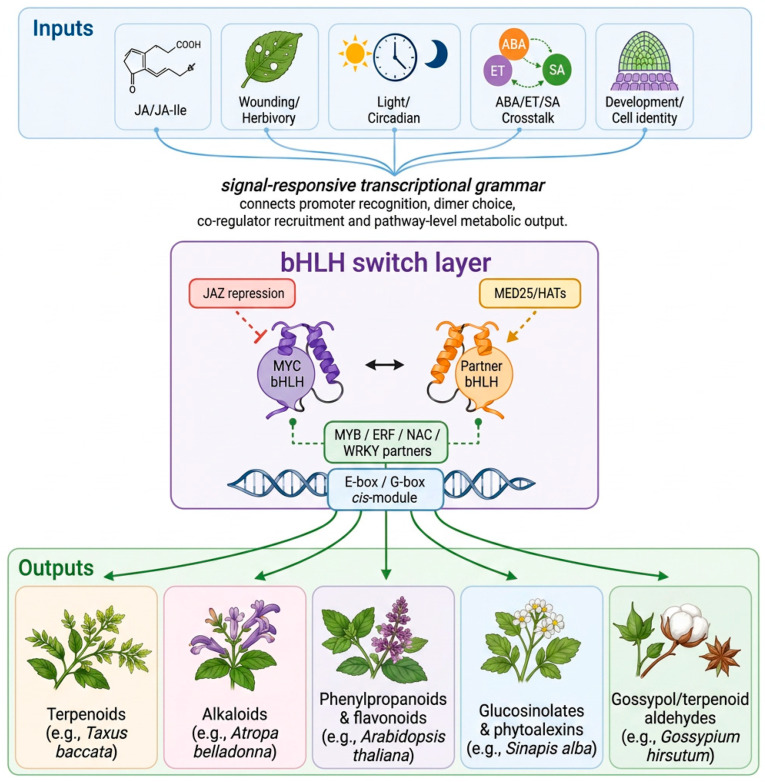
Conceptual framework for signal-responsive bHLH transcriptional grammar in plant specialized metabolism. Diverse inputs converge on a context-dependent bHLH regulatory layer that integrates promoter recognition, dimer selection, coregulator recruitment, and pathway-level metabolic output. Solid arrows indicate supported activation or signal flow; blunt-ended lines indicate repression; double-headed arrows indicate protein association or dimerization; and dashed arrows indicate inferred, indirect, or context-dependent regulation. The arrows summarize regulatory relationships and do not imply direct promoter binding unless explicitly stated. Boxed modules denote pathway branches, cellular contexts, or trait outputs. Abbreviations: JA, jasmonic acid; JA-Ile, jasmonoyl-L-isoleucine; ABA, abscisic acid; ET, ethylene; SA, salicylic acid; bHLH, basic helix-loop-helix; JAZ, JASMONATE ZIM-domain protein; MYC, myelocytomatosis transcription factor; MED25, Mediator complex subunit 25; HATs, histone acetyltransferases; MYB, myeloblastosis; ERF, ethylene response factor; NAC, NAM, ATAF1/2, and CUC2; WRKY, WRKY transcription factor; E-box/G-box, enhancer-box/G-box cis-regulatory elements. AI-tool disclosure: The diagram was generated using Banana 2 and subsequently checked and edited by the authors.

Within this regulatory landscape, basic helix-loop-helix (bHLH) transcription factors are well suited to function as signal-responsive control nodes. They recognize short DNA motifs, form dimers, respond to hormone-dependent repression or activation, and cooperate with other transcription-factor families. These properties enable a bHLH protein to coordinate sets of pathway genes rather than a single enzymatic step. In practical terms, a bHLH regulator can translate signals such as jasmonate, wounding, light, cell identity, or developmental transitions into pathway-level metabolic responses. This capacity makes bHLH regulators important both for understanding natural metabolic diversification and for designing more selective strategies for crop improvement and synthetic biology (Figure 1) [18,19,20,21,22,23,24,25,26,27,28,29,30,31,32,33,34,35,36,37,38,39,40,41].

A bHLH factor rarely functions as a universal master regulator in isolation. Its output is shaped by promoter grammar, partner proteins, chromatin accessibility, feedback regulation, and the competence of the cell in which it is expressed. The same MYC-like regulator may activate different metabolic branches in different tissues, whereas closely related bHLH paralogs may function as activators, antagonists, or developmental gates. Here, the term “bHLH switch” denotes a graded, context-dependent control node rather than a binary device or universal master regulator. Its state can range from repression through partial activation to full pathway output, depending on signal strength, partner availability, and cellular capacity. We first describe the structural and signaling logic that underlies bHLH switch behavior, using the jasmonate-JAZ-MYC module as the central example. We then compare bHLH-centered networks across terpenoids, alkaloids, phenylpropanoids and flavonoids, glucosinolates, phytoalexins, and cotton gossypol as a defense-toxicity case study. The final sections address antagonistic regulators, spatial specificity, engineering strategies, and standards of evidence.

To strengthen comparisons across species, this review distinguishes conserved regulatory grammar from lineage-specific implementation. Conserved features include JA/JA-Ile perception, JAZ-mediated repression, MYC/bHLH binding to E-box/G-box promoter motifs, coregulator recruitment, and feedback attenuation. By contrast, species-specific outputs arise from promoter architecture, partner-TF availability, chromatin accessibility, cellular storage capacity, growth–defense trade-offs, and the presence of activating or antagonistic bHLH paralogs. Accordingly, a bHLH validated in Arabidopsis, Taxus, Artemisia, Catharanthus, Salvia, Ginkgo, rice, or cotton should not be assumed to function equivalently in another species without evidence from direct binding, genetic perturbation, and metabolite-level analyses.

Unlike previous reviews that primarily summarize jasmonate signaling, MYC2 biology, or lists of transcription-factor–metabolite associations, this review presents some new perspectives. First, it classifies bHLH control as direct, cascade, or hybrid according to whether the factor binds structural-gene promoters, activates secondary transcription-factor tiers, or combines both routes. Second, it treats promoter grammar—the identity, number, spacing, and flanking context of E-box/G-box and partner-factor motifs—as a determinant of pathway specificity. Third, it integrates cell-type competence, precursor supply, transport, and storage anatomy into the interpretation of transcriptional effects. Fourth, it applies an evidence hierarchy that explicitly distinguishes intact-plant genetic, binding, and metabolite evidence from cultured-cell, transient, heterologous, co-expression, or predicted-motif evidence. Fifth, it identifies six cross-system principles—signal gating, topology matched to pathway architecture, partner-dependent promoter decoding, spatial competence, feedback rheostats, and evidence-dependent transferability—thereby establishing the bHLH-switch concept as a comparative and testable framework rather than merely a narrative metaphor.

### Review Methodology and Evidence Hierarchy

This narrative review was based on a structured literature search of the Web of Science Core Collection, Scopus, PubMed, and Google Scholar. The search was updated on 20 July 2026. Search terms combined “basic helix-loop-helix,” bHLH, MYC2, MYC3, or MYC4 with jasmonate, JA-Ile, or JAZ and with “specialized metabolism,” “secondary metabolism,” terpenoid*, alkaloid*, phenylpropanoid*, flavonoid*, glucosinolate*, phytoalexin*, gossypol, paclitaxel, or artemisinin. Targeted searches additionally included miRNA/microRNA, promoter grammar, cell type, trichome, gland, transport, and storage. Reference lists and citing articles of key primary studies and reviews were searched backward and forward.

Peer-reviewed English-language studies were included when they examined plant bHLH/MYC/JAZ regulators in relation to specialized-metabolite genes, metabolite accumulation, relevant cell types, or pathway-engineering outcomes. Studies were excluded if they focused on non-plant bHLHs, lacked a specialized-metabolism endpoint, duplicated an earlier report, or presented only unvalidated in silico predictions. Titles and abstracts were screened before full-text assessment, and studies were selected for mechanistic relevance rather than effect-size pooling. Conflicting findings were not averaged; instead, they were compared according to species, paralog, tissue or cell-culture system, elicitor dose and timing, assay type, and whether metabolite accumulation was measured.

Evidence was weighted across four levels: (1) strongest evidence—intact-plant loss- and gain-of-function genetics combined with direct promoter-occupancy or motif tests and quantitative metabolite phenotyping; (2) stable transgenic-plant, hairy-root, or cell-line perturbation supported by promoter and metabolite evidence; (3) transient or heterologous reporter, binding, or protein-interaction assays with expression readouts; and (4) supportive but non-causal evidence from co-expression, motif prediction, or network inference. Conclusions are framed according to the highest evidence level available, and intact-plant effects are not inferred solely from cultured-cell data.

## 2. Structural Grammar of bHLH Metabolic Switches

The bHLH domain comprises a basic DNA-binding region followed by two helices separated by a flexible loop. The basic region typically recognizes E-box elements containing the core sequence CANNTG and, in many cases, the G-box variant CACGTG. The HLH region mediates homo- or heterodimerization. Consequently, DNA binding, target preference, and transcriptional strength are not fixed properties of an individual monomer; rather, they emerge from the dimeric complex, its activation or repression domains, the surrounding promoter sequence, and the cellular context [42,43,44,45,46,47,48,49,50,51,52,53,54,55,56,57].

Regions outside the conserved bHLH domain provide an additional layer of switch control. MYC-type bHLHs contain JAZ-interaction domains that link them to jasmonate signaling. Flavonoid-associated bHLHs function in MYB-bHLH-WD40 complexes, whereas other family members interact with ERF, NAC, WRKY, bZIP, VQ-domain, or Mediator components. Some bHLHs act as activators, whereas others attenuate responses by sequestering partners, competing for DNA motifs, or recruiting repression machinery. Thus, the presence of a G-box in a promoter is not, by itself, evidence of a functional bHLH target. Productive activation also requires the appropriate bHLH dimer, accessible chromatin, neighboring cis-elements, coregulators, a permissive hormone state, and cell-type competence.

Combinatorial regulation with other TF families is central to this regulatory grammar. MYB proteins often confer branch and tissue specificity in flavonoid and phenylpropanoid pathways; WRKY and ERF factors frequently transmit JA-, pathogen-, or elicitor-associated signals to downstream specialized-metabolic branches; NAC factors can connect secondary metabolism with organ development and stress adaptation; and bZIP factors link specialized metabolism to ABA, light, and broader stress responses. These interactions indicate that bHLH regulators frequently operate within multi-TF circuits rather than as isolated master switches.

This regulatory grammar helps explain how broad regulators such as MYC2 generate distinct metabolic outputs in different tissues. In wounded Arabidopsis leaves, MYC proteins may enhance volatile sesquiterpene and glucosinolate defenses; in medicinal-plant roots, related MYC factors may activate tanshinone or phenolic-acid pathways; and in glandular trichomes, a MYC-centered module may influence both anatomical capacity and pathway flux. This flexibility is attractive for engineering but also entails risk: bHLH factors can increase pathway output, yet unrestricted activation may disrupt development or impose growth–defense trade-offs [58,59,60,61,62,63,64,65,66,67,68,69,70,71,72,73,74,75,76].

## 3. JA-JAZ-MYC2 as a Prototype Conditional ON/OFF Module

The jasmonic acid (JA)/jasmonate pathway provides the clearest example of a bHLH metabolic switch. When jasmonate levels are low, JAZ repressors bind MYC transcription factors and recruit NINJA-TOPLESS-associated repression machinery, maintaining target genes in a low-output state. Wounding, herbivory, pathogen challenge, or elicitor treatment enhances jasmonate signaling and promotes the formation of jasmonoyl-isoleucine (JA-Ile). JA-Ile drives assembly of the SCFCOI1-JAZ coreceptor complex, followed by JAZ ubiquitination and 26S-proteasome-mediated degradation. The released MYC factors then bind E-box/G-box motifs, recruit transcriptional machinery—including MED25-associated complexes—and activate primary target genes or secondary transcription-factor cascades [18,19,20,21,22,23,24,25,26,27,28,29,30,31].

**Figure 2:**
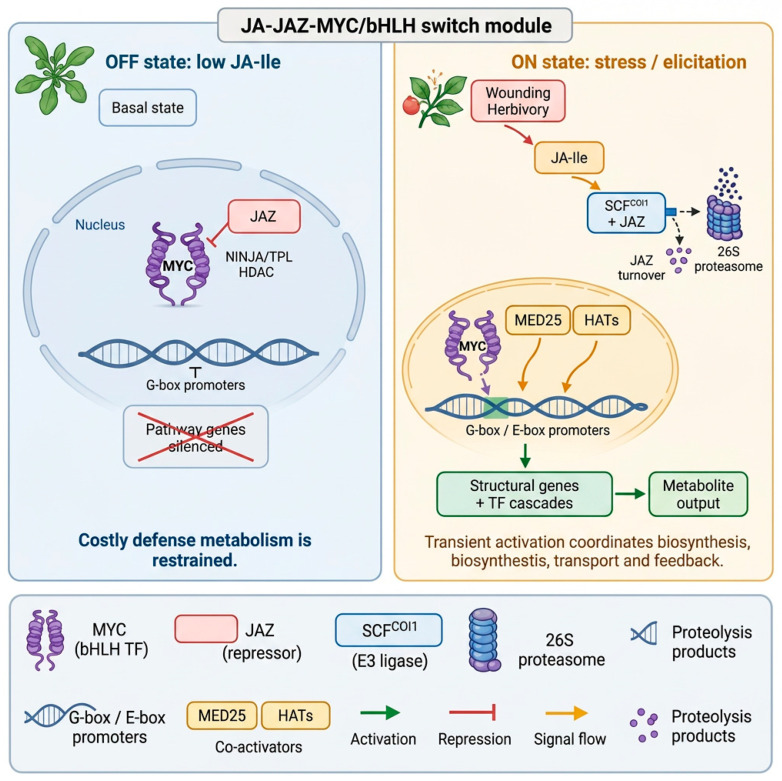
The JA-JAZ-MYC/bHLH conditional ON/OFF module. Low JA-Ile maintains JAZ-mediated repression, whereas stress or elicitation promotes SCF^COI1-dependent JAZ recognition and proteasomal turnover, thereby releasing MYC/bHLH activity. Solid green arrows indicate established activation or output flow; red blunt-ended lines indicate direct JAZ-mediated repression of MYC; yellow arrows indicate JA-Ile/SCF^COI1 signal flow; and dashed arrows toward the proteasome indicate ubiquitin-proteasome-dependent turnover. Coactivator arrows denote recruitment or activation rather than direct DNA binding. Abbreviations: JA, jasmonic acid; JA-Ile, jasmonoyl-L-isoleucine; JAZ, JASMONATE ZIM-domain protein; MYC, myelocytomatosis transcription factor; bHLH, basic helix-loop-helix; NINJA, NOVEL INTERACTOR OF JAZ; TPL, TOPLESS; HDAC, histone deacetylase; SCF, Skp1-Cullin-F-box complex; COI1, CORONATINE INSENSITIVE 1; MED25, Mediator complex subunit 25; HATs, histone acetyltransferases; TF, transcription factor; E-box/G-box, enhancer-box/G-box cis-regulatory elements. AI-tool disclosure: The diagram was generated using Banana 2 and subsequently checked and edited by the authors.

Although commonly depicted as an ON/OFF module, this pathway is not strictly binary. The ON/OFF representation is mechanistic shorthand for a graded and time-dependent response. JAZ proteins differ in their expression domains and degradation kinetics (Figure 2), whereas MYC proteins form multiple dimers and are regulated through phosphorylation, ubiquitination, and SUMOylation. MYC activity can also induce its own inhibitory mechanisms, including JAZ genes and antagonistic bHLH proteins, thereby preventing excessive defense activation. Light, the circadian clock, and other hormone pathways further modulate response timing and amplitude. Interactions with DELLA, EIN3/EIL1, NPR1, and ABA-related components help explain why the same elicitor can produce different metabolite profiles depending on tissue age, water status, pathogen type, or time of day [23,24,25,26,27,28,29,30,31,32,33,34,35,36,37,38,39].

In specialized metabolism, MYC factors act through both direct and indirect routes. They may bind promoters of structural genes while also activating ERF, MYB, NAC, WRKY, or other bHLH regulators that refine branch-specific expression. Many pathways appear to use a hybrid architecture in which a MYC factor enhances early precursor supply or key enzymatic steps, whereas downstream transcription factors distribute the signal among pathway branches. This architecture is particularly advantageous for long, branched pathways such as those producing taxanes, artemisinin, terpenoid indole alkaloids, and gossypol [36,37,38,39,40,41,58,59,60,61,62,63,64,65,66].

## 4. Metabolite Classes Governed by bHLH-Centered Networks

Cross-pathway comparisons indicate that pathway architecture, rather than metabolite class alone, determines whether bHLH control is direct, cascade-based, or hybrid. Compact defense branches with shared E-box/G-box-containing promoters can be coordinated directly, as in Arabidopsis terpene and glucosinolate responses. Long, branched pathways, including taxane and terpenoid indole alkaloid pathways, more often recruit ERF, BIS, MYB, or WRKY tiers to allocate flux among modules. Artemisia and Salvia additionally employ antagonistic bHLHs and trichome- or root-specific competence, demonstrating that activation and restraint can coexist within the same pathway [31,32,33,34,35,36,37,38,39,51,52,53,54,55,56,57,58,59,60,61,62,63,64,65,66,67,68,69,70,71,72,73].

Alkaloid regulation provides the clearest example of cascade control. In Catharanthus roseus, CrMYC2 activates ORCA factors, whereas BIS1/2 controls the iridoid branch; additional AP2/ERF, WRKY, MYB, GATA, and light-responsive factors coordinate branch-specific enzymes and cell-type programs. This multi-transcription-factor architecture is required because precursor generation, monoterpenoid synthesis, and indole coupling occur in distinct cellular contexts. It also explains why overexpression of a single upstream bHLH may increase selected transcripts without producing a proportional increase in final alkaloid accumulation [36,37,38,39,74,75,76,77,78,79,80,81,82].

Flavonoid and phenylpropanoid pathways illustrate obligatory partner dependence. TT8, GL3, and EGL3 do not specify pathway output alone: R2R3-MYBs confer branch and tissue identity, WD40 proteins stabilize the complex, and combinations of promoter motifs determine which structural genes respond. A bHLH may therefore function as an activator in one complex, as a competitor or partner-sequestering brake in another, or appear inactive when the required MYB/WD40 partner or permissive chromatin state is absent [40,41,42,43,44,45,46,47,48,49,50,83,84,85,86].

**Figure 3:**
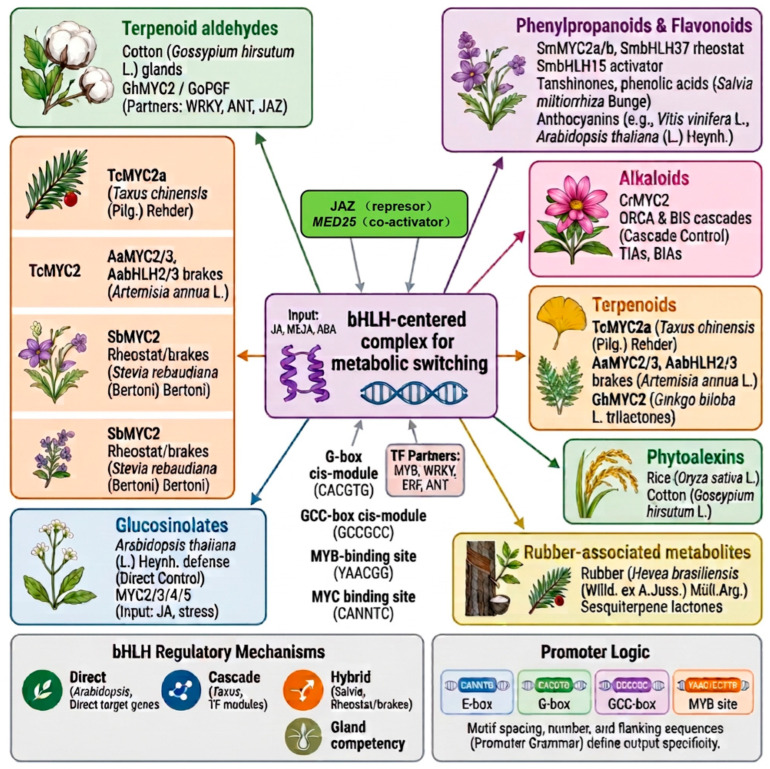
Context-dependent bHLH-centered regulatory hub for plant specialized metabolism. The schematic compares direct, cascade, and hybrid regulatory modes and illustrates how promoter grammar, partner transcription factors, and cell-type or gland competence shape pathway output. Gray arrows from JAZ, MED25, partner transcription factors, and cis-regulatory modules denote established repressor, coactivator, protein-partner, or promoter–input relationships. Colored arrows toward metabolite classes summarize cross-species regulatory associations and do not, by themselves, indicate direct promoter binding. The direct, cascade, hybrid, and gland-competence icons classify the principal regulatory mechanism and guide evidence interpretation. Solid arrows represent supported relationships; no proposed synthetic designs are shown. Abbreviations: bHLH, basic helix-loop-helix; MYC, myelocytomatosis transcription factor; JA, jasmonic acid; MeJA, methyl jasmonate; ABA, abscisic acid; JAZ, JASMONATE ZIM-domain protein; MED25, Mediator complex subunit 25; TF, transcription factor; MYB, myeloblastosis; WRKY, WRKY transcription factor; ERF, ethylene response factor; ANT, AINTEGUMENTA; GoPGF, Gossypium pigment gland formation; ORCA, octadecanoid-responsive Catharanthus AP2/ERF-domain transcription factor; BIS, bHLH iridoid synthesis transcription factor; TIA, terpenoid indole alkaloid; BIA, benzylisoquinoline alkaloid; E-box/G-box/GCC-box, enhancer-box/G-box/GCC-box cis-regulatory elements. AI-tool disclosure: The diagram was generated using Banana 2 and subsequently checked and edited by the authors.

Across systems, direct control is favored when the same bHLH can access several structurally related promoters within the relevant cell. Cascade control is favored when a long or compartmented pathway requires branch-specific regulators, whereas hybrid control is favored when an upstream bHLH must both increase precursor supply and delegate downstream specificity. Whether a bHLH activates or represses its targets depends on its activation or repression domains, dimer choice, JAZ affinity, coactivator or corepressor recruitment, motif spacing and flanking sequence, chromatin accessibility, and competition with antagonistic paralogs. Figure 3 summarizes this direct–cascade–hybrid framework.

### 4.1. Cross-Pathway Comparison and Limiting Factors

Transcriptional activation may fail to increase metabolite levels when enzymes are substrate-limited, pathway steps are imbalanced, products are rapidly turned over, or transport and storage capacities are saturated. Specialized structures—including glandular trichomes, secretory cells, vacuoles, resin ducts, and cotton pigment glands—can therefore set the upper limit of a transcriptional intervention. Toxic intermediates and growth–defense costs may also activate feedback mechanisms that attenuate the response. Consequently, transcript induction, promoter binding, and metabolite accumulation should be treated as distinct evidence layers rather than interchangeable endpoints [58,59,60,61,62,63,64,65,66,67,68,69,70,71,72,73,74,75,76,86,87,88,89,90,91,92,93,94,95,96,97,98,99,100,101,102,103,104,105,106,107,108,109,110].

The context in which evidence is generated is equally important. Conclusions concerning Arabidopsis glucosinolate and flavonoid pathways are supported by intact-plant genetics and metabolite phenotypes, whereas many claims in medicinal plants derive from suspension cells, hairy roots, transient assays, or elicitor treatments. These systems are valuable for mechanistic discovery but may lack native developmental gradients, transport routes, storage anatomy, or whole-plant trade-offs. We therefore identify cultured-cell evidence explicitly and avoid extrapolating such findings to intact plants without stable genetic and metabolite-level validation.

Broader reviews and comparative studies place bHLH regulation within plant development, stress responses, and secondary-metabolite control [111,112,113,114,115]. Related work spans bioactive-metabolite diversity, extraction and analytics, synthetic biology, microbiome and metabolic engineering, land-plant evolution, cotton phytoalexins, ecological interactions, quantitative cell biology, flavonoid regulation, trichome development, light signaling, circadian control, and abiotic stress [116,117,118,119,120,121,122,123,124,125,126,127,128,129,130,131,132,133]. Hormone-network integration is further discussed by Rafiq et al. [134].

### 4.2. Contradictory and Context-Dependent Evidence

Taxus illustrates why apparently opposing findings should not be forced into a single model. In Taxus cuspidata suspension cells, transient overexpression of TcJAMYC1, TcJAMYC2, and TcJAMYC4 repressed several methyl-jasmonate-responsive paclitaxel-promoter reporters, supporting a negative or feedback role [56]. By contrast, in Taxus chinensis suspension cells, TcMYC2a interacted with TcJAZ3, activated T/G-, G-, and E-box reporters, increased multiple taxane-pathway transcripts, induced TcERF15, and enhanced paclitaxel accumulation after stable overexpression, supporting positive direct-plus-cascade control [55].

These findings are not necessarily mutually exclusive. The studies examined different Taxus species, MYC paralogs, cell lines, transformation formats, promoter panels, and sampling windows. Because MeJA responses are transient, a MYC factor may contribute to early activation in one context and to later attenuation in another. Moreover, both studies used cultured cells rather than intact trees. The most defensible conclusion is therefore that Taxus MYC-like bHLHs are paralog-, phase-, and context-dependent regulators. Their net effects should be resolved through matched time courses, loss-of-function alleles, promoter-occupancy assays, and taxane measurements conducted in the same genetic and cellular background.

Similar caution applies to activator–antagonist pairs in Artemisia and Salvia and to cotton modules in which pathway transcription is genetically coupled to gland development. When studies disagree, priority should be given to evidence that combines direct binding, genetic perturbation, and metabolite phenotyping in the relevant tissue. Co-expression or reporter activity alone should be regarded as hypothesis-generating evidence.

### 4.3. Common Regulatory Principles Across Species and Metabolite Classes

The comparisons above reveal six recurring principles that extend across species and metabolite classes. First, bHLH output is signal gated rather than constitutive, most commonly through the JA-JAZ-MYC module but also through light, developmental, ABA, ET, and SA inputs. Second, network topology follows pathway architecture: compact or clustered pathways tend to favor direct control, long and compartmented pathways tend to favor cascades, and many systems use hybrid control. Third, promoter grammar and partner transcription factors determine branch specificity; a G-box alone is insufficient. Fourth, chromatin accessibility, cell identity, precursor supply, transport, and storage establish a competence ceiling for transcriptional activation. Fifth, antagonistic paralogs and feedback loops convert apparent ON/OFF behavior into a graded rheostat. Sixth, the transferability of a candidate regulator depends on the evidence context; conclusions from transient or cultured-cell assays should not be generalized across species without matched genetic, binding, and metabolite validation [18,19,20,21,22,23,24,25,26,27,28,29,30,31,32,33,34,35,36,37,38,39,40,41,42,43,44,45,46,47,48,49,50,55,56,57,58,59,60,61,62,63,64,65,66,67,68,69,70,71,72,73,74,75,76,77,78,79,80,81,82,83,84,85,86,87,88,89,90,91,92,93,94,95,96,97,98,99,100,101,102,103,104,105,106,107,108,109,110].

Together, these principles refine the bHLH switch as a multilayered regulatory framework comprising (i) signal release, (ii) dimer and partner selection, (iii) promoter decoding, (iv) spatial and metabolic competence, and (v) feedback-limited output. The framework predicts that engineering success will be greatest when the targeted regulatory layer matches the pathway bottleneck: promoter or activator editing for direct-control systems, secondary-TF tuning for cascade systems, and tissue-specific or storage engineering for competence-limited systems. Table 1 summarizes this cross-system synthesis.

### 4.4. miRNA-Mediated Regulation of bHLH Transcription Factors

bHLH switch activity is also regulated post-transcriptionally by microRNAs (miRNAs), which are typically 20–24-nucleotide regulatory RNAs that guide ARGONAUTE-containing RNA-induced silencing complexes to complementary transcripts, causing transcript cleavage and/or translational repression. Because transcription-factor abundance can be rate limiting, miRNA-target interactions can sharpen developmental, tissue-specific, and stress-responsive outputs and can form feedback or feed-forward loops with transcription factors [135]. Direct regulation of bHLH transcripts has been experimentally demonstrated in several plant lineages. In Arabidopsis, miR396a reduces bHLH74 transcript abundance; genetic analyses of mir396a, bhlh74, and miR396-resistant bHLH74 lines established that this module controls root elongation [136]. In Marchantia polymorpha, the lineage-specific MpFRH1 miRNA directly targets the RSL class I bHLH MpRSL1, forming a negative-feedback circuit that controls rhizoid differentiation [137]. These examples demonstrate that small RNAs can directly gate bHLH dosage, although direct miRNA targeting of the specialized-metabolism bHLHs discussed above remains less extensively validated.

miRNAs may also regulate bHLH-centered metabolic switches indirectly by altering the availability of interacting partners or the stability of multimeric complexes. In Arabidopsis, miR156 targets SQUAMOSA PROMOTER BINDING PROTEIN-LIKE (SPL) factors; SPL9 can destabilize the MYB-bHLH-WD40 complex and thereby redirect flux between anthocyanin and flavonol branches [138]. The miR828-TAS4 pathway generates trans-acting small interfering RNAs that target anthocyanin-associated MYB partners, adding another small-RNA regulatory layer to the bHLH-containing MBW complex [139]. Thus, miRNA networks may tune bHLH-dependent metabolism through both direct control of bHLH transcripts and indirect control of partner transcription factors. Candidate miRNA-bHLH relationships inferred from small-RNA sequencing, degradome data, or co-expression should be validated by cleavage-site mapping, reporter or sensor assays, miRNA-resistant target alleles, and metabolite-level phenotyping before being used for engineering. Representative bHLH factors, evidence contexts, and limitations are summarized in Table 2.

## 5. Cotton Gossypol as an In-Depth Case Study of Spatial Competence and Defense-Toxicity Trade-Offs

Cotton is presented as an intentionally detailed case study, not as a claim that this system is more universal than others. It was selected because it links JA-JAZ-MYC pathway regulation, bHLH-dependent gland development, cell-type-specific storage anatomy, defense value, seed toxicity, and a concrete tissue-specific engineering objective within a single system. Gossypol and related terpenoid aldehydes accumulate in pigment glands and contribute to resistance against insects and pathogens. However, high gossypol concentrations in seeds restrict the use of cottonseed protein and oil in food and in feed for monogastric animals. The breeding objective is therefore not simply to increase or decrease gossypol, but to reduce seed toxicity while preserving vegetative defense [86,87,88,89,90,91,92,93,94,95,96,97,98,99,100].

The pathway begins with farnesyl diphosphate and proceeds through (+)-delta-cadinene, hydroxylated intermediates, hemigossypol, and dimerized products. Key genes include CDNC, CYP706B1, DH1, CYP82D113, and CYP71BE79. GhMYC2 binds G-box elements in the promoters of gossypol-biosynthetic genes and activates CYP71BE79 and related pathway components, whereas GhJAZ2 inhibits this activation, placing the system within the JA-JAZ-MYC framework. GoPGF adds a second bHLH-centered layer by coupling pigment-gland development to gossypol biosynthesis. Recent single-cell and regulatory-network studies further suggest that gland-specific transcriptional hierarchies, feedback loops, and WRKY- or ANT-associated cascades determine where and to what extent terpenoid aldehydes accumulate [90,91,92,93,94,95,96,97,98,99].

**Figure 4:**
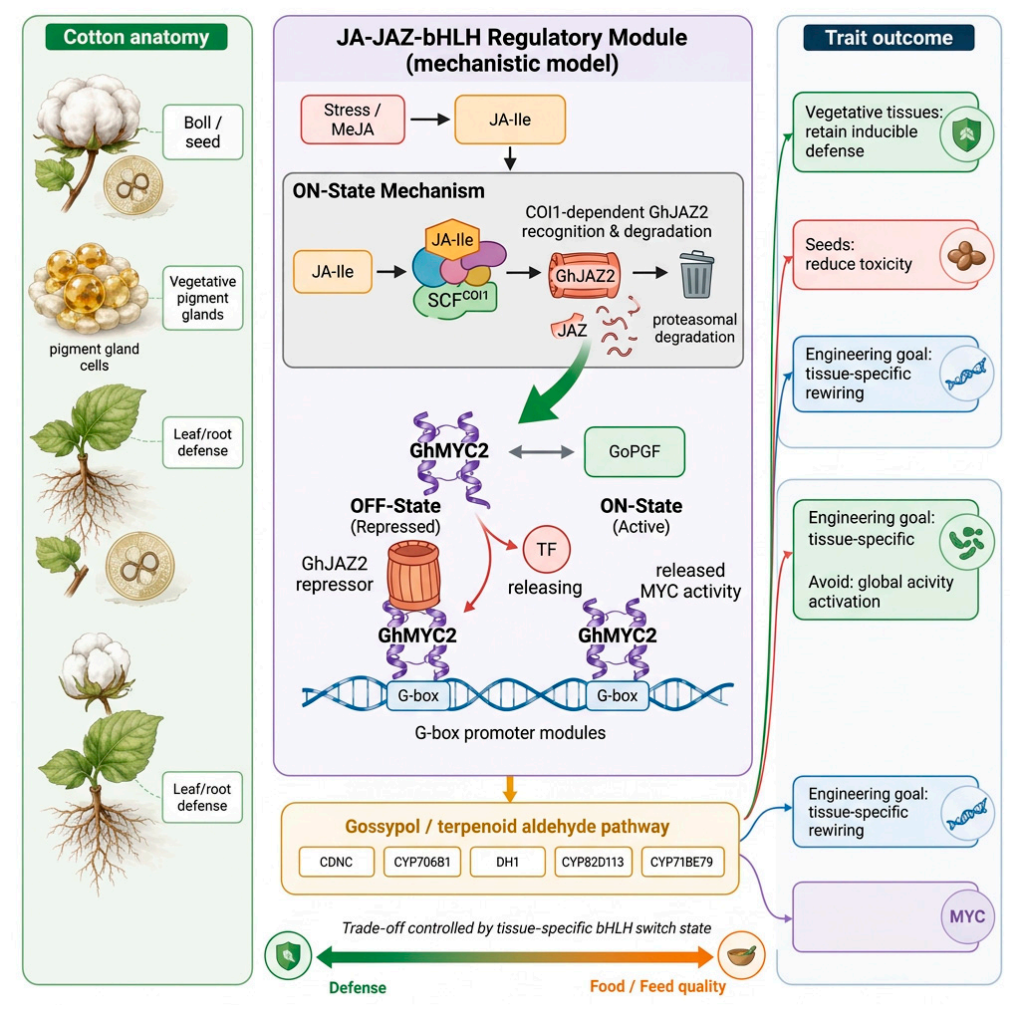
Cotton JA-JAZ-GhMYC2/GoPGF regulatory core controlling gossypol and terpenoid aldehydes. Stress or MeJA increases JA-Ile levels, promoting SCF^COI1-dependent recognition and proteasomal degradation of GhJAZ2 and thereby releasing GhMYC2 activity. GoPGF and pigment-gland competence couple pathway transcription to tissue-specific storage and defense-toxicity trade-offs. Solid black or green arrows indicate established signaling or activation steps; red blunt-ended lines indicate direct repression; the double-headed arrow between GhMYC2 and GoPGF indicates regulatory coupling and does not necessarily imply direct physical binding; and arrows toward trait boxes represent conceptual tissue-level or engineering outcomes rather than direct molecular interactions. Abbreviations: JA, jasmonic acid; JAZ, JASMONATE ZIM-domain protein; Gh, Gossypium hirsutum; MYC2, myelocytomatosis 2 transcription factor; GoPGF, Gossypium pigment gland formation; MeJA, methyl jasmonate; JA-Ile, jasmonoyl-L-isoleucine; SCF, Skp1-Cullin-F-box complex; COI1, CORONATINE INSENSITIVE 1; TF, transcription factor; CDNC, (+)-delta-cadinene synthase; CYP, cytochrome P450; DH1, short-chain alcohol dehydrogenase 1; G-box, G-box cis-regulatory element. AI-tool disclosure: The diagram was generated using Banana 2 and subsequently checked and edited by the authors.

The cotton example reinforces a broader principle: an effective bHLH switch should not merely increase metabolite production; its activity must also be spatially constrained. Seed-specific suppression can improve nutritional quality, whereas broad suppression may compromise defense. Conversely, ectopic activation of GhMYC2 or GoPGF could increase toxicity or impose additional growth costs. A more effective strategy is tissue-aware rewiring that preserves protective aldehydes in vegetative tissues and pigment glands while reducing toxic accumulation in seed tissues (Figure 4).

## 6. Engineering Principles for Breeding and Synthetic Biology

MYC2 is central to jasmonate-responsive metabolism, but it represents only one component of a much larger bHLH family. Non-MYC bHLHs—including TT8-like flavonoid regulators, BIS alkaloid regulators, rice DPF, cotton GoPGF, and lineage-specific medicinal-plant regulators—can activate, repress, partition, or gate metabolic flux. Antagonistic bHLHs merit particular attention because inducible defense pathways require effective braking mechanisms. AabHLH2/3 counteract AaMYC2 during artemisinin regulation, SmbHLH37 antagonizes SmMYC2 in *Salvia* phenolic-acid metabolism, and cotton feedback modules restrain gland and phytoalexin output. These antagonists are not merely obstacles; they may provide useful tools for tuning branch specificity and reducing pleiotropy [52,61,62,86,87,88,89,90,91,92,93,94,95,96,97,98,99].

Lineage specificity commonly arises through gene duplication followed by regulatory rewiring. One paralog may retain broad jasmonate responsiveness, whereas another acquires tissue-specific expression, altered JAZ affinity, or a distinct target-promoter repertoire. Comparative genomics, co-expression analysis, metabolomics, and promoter-motif analysis are valuable for nominating candidate switches, but functional assignment still requires genetic perturbation, promoter-binding assays, motif mutagenesis, protein-interaction assays, and metabolite measurements. Annotation alone is insufficient, particularly because some bHLH-like proteins have atypical DNA-binding capacity yet still regulate metabolism through partner sequestration or control of protein stability [101,102,103,104,105].

Multi-omics approaches are accelerating the discovery of bHLHs and their partner TFs. Bulk, comparative, and time-series transcriptomics can identify TFs responsive to JA, stress, tissue identity, or development; single-cell and spatial transcriptomics can assign regulatory candidates to glands, trichomes, vascular tissues, seed compartments, or specific root cell types; and epigenomic approaches such as ATAC-seq, ChIP-seq, and CUT&Tag can reveal accessible promoters and direct binding sites. Proteomics, phosphoproteomics, and interactomics can detect JAZ, MED25, MYB, WRKY, NAC, ERF, bZIP, and chromatin-associated partners, whereas targeted or untargeted metabolomics can connect regulatory perturbations to pathway output. AI-assisted regulatory-network prediction can integrate co-expression, motif grammar, chromatin accessibility, protein-interaction, and metabolite features to prioritize candidates.

bHLH switches are attractive engineering targets because a single regulator can coordinate multiple enzyme genes and secondary regulators. This breadth, however, is also their principal risk. Constitutive MYC activation can redirect carbon, nitrogen, ATP, and reducing power away from growth and yield, while hormone crosstalk may alter drought tolerance, senescence, pathogen resistance, or flowering. Successful engineering must therefore constrain bHLH output in space, time, and amplitude [68,69,70,71,72,73,74,75,76,106,107,108,109,110,111,112].

**Figure 5:**
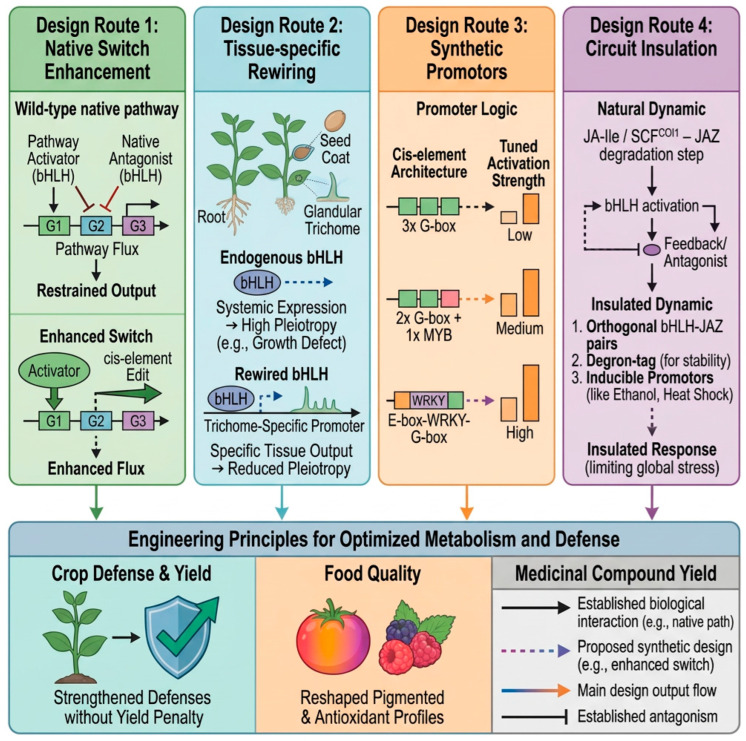
Engineering strategies for tuning bHLH-regulated metabolism while limiting growth and defense costs. Route 1 enhances native activators or edits cis-elements; Route 2 confines bHLH activity to selected tissues; Route 3 applies synthetic promoter grammar; and Route 4 insulates the native JA-Ile/SCF^COI1-JAZ degradation dynamics. Solid black arrows denote established biological interactions; blunt-ended black lines denote established antagonism; purple dashed arrows denote proposed synthetic designs; and colored arrows denote the primary design-to-output flow. Proposed engineering relationships are therefore visually distinguished from established mechanisms. Abbreviations: bHLH, basic helix-loop-helix; JA-Ile, jasmonoyl-L-isoleucine; SCF, Skp1-Cullin-F-box complex; COI1, CORONATINE INSENSITIVE 1; JAZ, JASMONATE ZIM-domain protein; MYB, myeloblastosis; WRKY, WRKY transcription factor; E-box/G-box, enhancer-box/G-box cis-regulatory elements. AI-tool disclosure: The diagram was generated using Banana 2 and subsequently checked and edited by the authors.

Four design routes are particularly promising. First, native-switch enhancement can involve overexpressing a pathway-specific activator, weakening a tissue-specific antagonist, or editing cis-elements in structural-gene promoters. Second, tissue-specific rewiring can direct bHLH activity to trichomes, glands, roots, or seed coats, thereby limiting systemic pleiotropy. Third, synthetic promoters can combine selected numbers and positions of G-box, E-box, MYB, ERF, or WRKY motifs to tune activation strength and regulatory logic. Fourth, circuit insulation can use modified JAZ-bHLH interactions, degrons, inducible promoters, or orthogonal transcriptional modules to reproduce natural switch dynamics while reducing global stress responses (Figure 5).

Future engineering strategies may include CRISPR/Cas-mediated knockout of repressors, promoter editing of E-box/G-box and MYB/WRKY/ERF cis-elements, base or prime editing of regulatory residues, and multiplex tuning of paralogous bHLH families. Synthetic promoters can combine defined numbers and spacings of hormone-, tissue-, and stress-responsive motifs to generate graded or logic-gated responses. Synthetic-biology platforms can also reconstruct minimal bHLH-JAZ-promoter modules in plants, hairy roots, cell cultures, or heterologous hosts. These strategies should be integrated with precursor-supply optimization, enzyme balancing, transport and storage engineering, and inducible or tissue-specific control to minimize pleiotropy.

In crops, these principles could strengthen inducible defense while limiting yield penalties. For food-quality traits, editing bHLH promoters or MBW components could reshape pigmentation and antioxidant profiles. In medicinal plants, bHLH activation should be coordinated with precursor supply, enzyme balancing, transport, and storage engineering. In microbial platforms, native plant bHLH modules may require substantial redesign because jasmonate perception, chromatin organization, and coregulators differ from those in plant cells.

### Translational Applications in Crops, Pharmaceuticals, and Stress Resilience

Translational applications fall into three broad categories. In crop improvement, bHLH modules may be used to strengthen inducible defense, tune glucosinolate or phenolic profiles, improve pigmentation and antioxidant quality, and reduce seed toxicity while preserving vegetative protection. In pharmaceutical-metabolite production, bHLH-centered modules provide entry points for increasing taxanes, artemisinin, terpenoid indole alkaloids, tanshinones, and other high-value metabolites when activator deployment is coordinated with precursor supply, enzyme stoichiometry, transport, and storage. In stress-resilient plants, inducible or tissue-specific bHLH control may help balance growth–defense trade-offs under drought, salinity, pathogen attack, or herbivory. Before field or industrial deployment, candidate designs should be evaluated for yield penalties, ecological effects, metabolite safety, regulatory compliance, and stability across environments.

## 7. Conclusions

bHLH transcription factors are among the most versatile regulatory switches in plant specialized metabolism. Their regulatory capacity derives from DNA-motif recognition, dimerization, hormone-responsive repression, combinatorial partner interactions, chromatin recruitment, feedback control, and cell-type competence. MYC2 and related MYC factors form the best-characterized jasmonate-responsive module, but the wider family also includes MBW flavonoid regulators, BIS alkaloid regulators, DPF phytoalexin regulators, cotton GoPGF/GhMYC2 modules, and numerous lineage-specific activators and antagonists.

The central conclusion is not that a single bHLH acts as a universal master regulator. Rather, plant lineages have repeatedly rewired a conserved bHLH regulatory grammar to control distinct chemical innovations. In the expanded framework developed here, signal release, dimer and partner selection, promoter decoding, spatial and metabolic competence, and feedback-limited output form interacting control layers that generate direct, cascade, or hybrid regulation. The direct–cascade–hybrid classification, together with the concepts of promoter grammar and cell-type competence, enables comparison across systems without obscuring their differences. Understanding this regulatory grammar should help researchers move from descriptive candidate genes toward precise engineering of valuable metabolites, stronger defenses, and reduced toxicity. The most effective designs will integrate bHLH switch biology with promoter grammar, evidence strength, cell-type specificity, precursor supply, transport, storage capacity, pathway balance, and ecological considerations. A practical next step is to combine comparative genomics, transcriptomics, epigenomics, proteomics, metabolomics, single-cell sequencing, AI-guided network inference, CRISPR-enabled regulatory editing, synthetic promoters, and pathway-level phenotyping to engineer bHLH modules with species-, tissue-, and trait-specific precision. Future strategies should also account for conserved or lineage-specific miRNA target sites and small-RNA control of bHLH partner proteins, because these post-transcriptional layers may influence tissue specificity, response amplitude, and metabolic trade-offs.

## Figures and Tables

**Table 1:** Cross-species synthesis of the shared regulatory principles that define context-dependent bHLH switches in plant specialized metabolism.

Regulatory Dimension	Conserved Comparative Principle	Representative Systems and Metabolite Classes	Key Context Dependence and Testable Implication
Signal gating	Recurrent JA-Ile-COI1-JAZ signaling releases MYC/bHLH activity, while light, developmental cues, and other hormones modulate response amplitude.	Arabidopsis terpenes and glucosinolates; Taxus taxanes; Artemisia artemisinin; cotton gossypol.	Use matched dose–response and time-course experiments and distinguish among JAZ/MYC paralogs; elicitation should not be treated as a fixed binary state.
Network topology	Direct, cascade, and hybrid regulatory modes are associated with pathway length, branching, and compartmentation.	Direct: Arabidopsis defense branches; cascade: Catharanthus terpenoid indole alkaloids; hybrid: Taxus, Salvia, and cotton.	Map both structural-gene targets and secondary-TF tiers; transcript induction alone may not predict final metabolite output.
Promoter and partner logic	E-box/G-box recognition depends on motif context, dimer selection, and combinatorial transcription-factor partners.	Flavonoid MYB-bHLH-WD40 complexes; ERF, WRKY, MYB, NAC, and bZIP partnerships in medicinal plants.	Test motif number, spacing, flanking sequence, partner dependence, and chromatin occupancy rather than relying on motif prediction alone.
Spatial and metabolic competence	Cell identity, chromatin accessibility, precursor supply, transport, and storage capacity define the ceiling of transcriptional activation.	Artemisia glandular trichomes; Salvia roots; cotton pigment glands; compartmented Catharanthus pathways.	Use tissue-specific perturbation and metabolite-localization analyses; broad overexpression can obscure the true limiting layer.
Feedback and antagonism	JAZ resynthesis, antagonistic bHLHs, and competition among paralogs convert apparent ON/OFF behavior into graded rheostats.	AaMYC2 versus AabHLH2/3; SmMYC2 versus SmbHLH37; contrasting Taxus MYC-like paralogs.	Resolve phase-, paralog-, and tissue-specific effects through matched loss- and gain-of-function analyses.
Evidence and engineering	Regulatory claims and engineering strategies should be scaled to both the strength of the evidence and the relevant pathway bottleneck.	Strong intact-plant evidence in several Arabidopsis, rice, and cotton systems versus cultured-cell evidence in Taxus and some medicinal plants.	Prioritize direct binding, genetic perturbation, and metabolite phenotyping; combine TF tuning with precursor, transport, and storage engineering.

**Table 2:** Representative bHLH transcription factors involved in plant specialized metabolism, including their principal evidence contexts and limitations.

bHLH TF/Module	Species	Target Metabolite/Pathway	Upstream Signals, Partners, Downstream Targets, Evidence Context, and Key Limitations
MYC2/MYC3/MYC4	Arabidopsis thaliana	Sesquiterpenes and glucosinolates	JA/JA-Ile, JAZ, DELLA, and MED25 shape direct or hybrid regulation of terpene-synthase and glucosinolate genes; outputs vary with tissue age, light conditions, and stress timing.
TcMYC2a; TcJAMYC1/2/4	Taxus chinensis and T. cuspidata (cultured cells)	Taxanes/paclitaxel-related metabolism	MeJA-responsive MYC-like factors show conflicting positive and negative effects. TcMYC2a supports direct-plus-ERF-cascade activation, whereas TcJAMYC1/2/4 repress promoter reporters. Differences in paralog, species, cell line, assay format, and timing remain unresolved, and intact-plant validation is lacking [55,56].
AaMYC2/AaMYC3; AabHLH2/3	Artemisia annua	Artemisinin	JA/MeJA signaling and glandular-trichome competence promote ADS, CYP71AV1, DBR2, and ALDH1 expression, whereas AabHLH2/3 illustrate antagonistic pathway tuning.
CrMYC2; BIS1/2	Catharanthus roseus	Terpenoid indole alkaloids	JA activates CrMYC2-, ORCA-, and BIS-centered cascades that coordinate the monoterpenoid and indole branches; cell-type and chromatin context likely refine pathway output.
SmMYC2a/b; SmbHLH37	Salvia miltiorrhiza	Tanshinones and phenolic acids	MeJA-induced SmMYC2 activators are counterbalanced by SmbHLH37; MYB and bZIP partners may help distinguish phenolic-acid from terpenoid branches.
TT8/GL3/EGL3 and related bHLHs	Arabidopsis, fruits, flowers and seeds	Anthocyanins and proanthocyanidins	MBW complexes integrate MYB, bHLH, and WD40 partners to regulate DFR, BAN, and related genes; promoter variation can rewire pigmentation.
GbMYC2	Ginkgo biloba	Terpene trilactones	JA/MeJA-responsive GbMYC2 activity is associated with terpene-trilactone biosynthesis, but direct targets require validation across developmental stages.
DPF-like bHLHs	Rice	Diterpenoid phytoalexins	Elicitor- and JA-responsive bHLH modules cooperate with defense signaling to regulate diterpene-biosynthetic gene clusters.
GhMYC2 and GoPGF	Gossypium spp.	Gossypol/terpenoid aldehydes	JA/MeJA, GhJAZ2, WRKY/ANT factors, and gland identity regulate CDNC, CYP706B1, CYP71BE79, and related genes; tissue-specific rewiring is needed to balance defense with seed quality.

## Data Availability

No new data were created or analyzed in this review; therefore, data sharing is not applicable.

## Abbreviations

ABA	abscisic acid
ATAC-seq	assay for transposase-accessible chromatin using sequencing
bHLH	basic helix-loop-helix
ChIP	chromatin immunoprecipitation
COI1	CORONATINE INSENSITIVE 1
CRISPR	clustered regularly interspaced short palindromic repeats
CUT&Tag	cleavage under targets and tagmentation
E-box	enhancer box cis-regulatory element
ERF	ethylene response factor
G-box	G-box cis-regulatory element
JA	jasmonic acid
JA-Ile	jasmonoyl-L-isoleucine
MBW	MYB-bHLH-WD40 regulatory complex
MPRA	massively parallel reporter assay
MYC	myelocytomatosis transcription factor
NAC	NAM, ATAF1/2, and CUC2 transcription factor
SCF	Skp1-Cullin-F-box E3 ubiquitin ligase complex
TF	transcription factor
WRKY	WRKY transcription factor
Y1H	yeast one-hybrid
AI	artificial intelligence
bZIP	basic leucine zipper transcription factor
HAT	histone acetyltransferase
JAZ	JASMONATE ZIM-domain protein
MeJA	methyl jasmonate
TFs	transcription factors
AGO	ARGONAUTE protein
ADS	amorpha-4,11-diene synthase
ALDH1	aldehyde dehydrogenase 1
ANT	AINTEGUMENTA transcription factor
AP2	APETALA2 transcription factor
BAN	BANYULS (anthocyanidin reductase)
BIA	benzylisoquinoline alkaloid
BIS	bHLH iridoid synthesis transcription factor
CDNC	(+)-delta-cadinene synthase
CYP	cytochrome P450
DBR2	artemisinic aldehyde delta-11(13) reductase 2
DELLA	DELLA-domain growth-repressor protein
DFR	dihydroflavonol 4-reductase
DH1	short-chain alcohol dehydrogenase 1
DPF	diterpenoid phytoalexin factor
EGL3	ENHANCER OF GLABRA3
EMSA	electrophoretic mobility shift assay
ET	ethylene
GCC-box	GCC-box cis-regulatory element
GL3	GLABRA3
GoPGF	Gossypium pigment gland formation
HDAC	histone deacetylase
MED25	Mediator complex subunit 25
miRNA	microRNA
NINJA	NOVEL INTERACTOR OF JAZ
ORCA	octadecanoid-responsive Catharanthus AP2/ERF-domain transcription factor
RISC	RNA-induced silencing complex
RSL	ROOT HAIR DEFECTIVE SIX-LIKE
SA	salicylic acid
siRNA	small interfering RNA
SPL	SQUAMOSA PROMOTER BINDING PROTEIN-LIKE
TAS4	TRANS-ACTING siRNA GENE 4
TIA	terpenoid indole alkaloid
TPL	TOPLESS co-repressor
TT8	TRANSPARENT TESTA 8
TTG1	TRANSPARENT TESTA GLABRA 1
WD40	WD40-repeat protein
